# Targeting FHL2 for EGFRvIII-positive glioblastoma

**DOI:** 10.18632/oncotarget.26422

**Published:** 2018-12-04

**Authors:** Lili Sun, Qing Lan, Ming Li

**Affiliations:** Ming Li: The Experimental Center, Department of Neurosurgery, The Second Affiliated Hospital of Soochow University, Suzhou, Jiangsu, China

**Keywords:** FHL2, EGFR, EGFRvIII, glioblastoma

Glioblastoma multiforme (GBM) is the most common and lethal malignant brain tumor with highly infiltrative and heterogeneous features, which hampers the current standard treatment including surgical resection, radiotherapy followed by temozolomide chemotherapy. As a result, the median survival time of GBM patients remains 12-16 months [1]. Based on The Cancer Genome Atlas data, GBM can be classified into 4 major molecular subgroups: proneural, neural, classical and mesenchymal. The classical subgroup is characterized by loss of the Ink4a/ARF and amplification/mutation of epidermal growth factor receptor (EGFR). EGFR is frequently amplified or mutated, or both in nearly 60% of GBM. EGFRvIII, an extracellular domain truncation from exons 2-7, is the most common mutation form of EGFR, which is found in 20-30% of GBM. Without binding to its any known ligand, EGFRvIII is constitutively active in GBM and plays a key role in tumorigenicity, invasion, and therapeutic resistance [2-3]. EGFRvIII preferentially activates the PI3K/Akt signaling pathway which is thought to contribute to its radiation resistance. EGFRvIII is exclusively expressed on the cell surface of GBMs and other tumors, but it is absent in normal tissues. Therefore, it is an ideal therapeutic target for GBM treatment. There are various ways to target EGFRvIII, including CAR T-cell therapy, therapeutic vaccines, antibodies, and small molecule inhibitors. However, these treatments are not as effective as expected.

The Four-and-a-half LIM (FHL)-only subfamily consists of five members: FHL1, FHL2 (also known as DRAL or SLIM3), FHL3, FHL4 and ACT (only present in the testis) [4]. The LIM domain contains a double-zinc finger motif that is known to exist in a variety of proteins, such as homeodomain transcription factors, kinases, and adaptors. LIM domain functions as an enzymatically inactive protein-protein interaction motif, through which it is capable of recognizing a plethora of downstream targets [4]. Amongst the different FHL proteins, FHL2 is the best studied one within the subfamily. FHL2 is mainly expressed in heart, but it is also found in kidney, lung, ovary, prostate, stomach, colon and brain. The FHL2 gene in human is mapped in the region of the chromosome 2q12-q14. FHL2 has been shown to participating in transcriptional regulation, cytoskeleton organization, cell lineage specification and organ development [4].

FHL2 expression and function varies significantly between different types of cancer. It may act as a tumor suppressor or promoter depending on the type of cancer [5, 6]. We previously provided the first evidence that FHL2 plays an oncogenic role in glioma. We demonstrated that FHL2 is not only detected in brain tissue, but it is also upregulated in both glioblastoma cell lines and human GBM specimens. Targeting FHL2 with RNA interference inhibits glioblastoma cell proliferation and migration. Conversely, upregulating FHL2 expression in glioma cell lines stimulated proliferation, anchorage-independent growth, migration and tumorigenicity in mice [7]. However, the underlying mechanisms are not clear.

**Figure 1:**
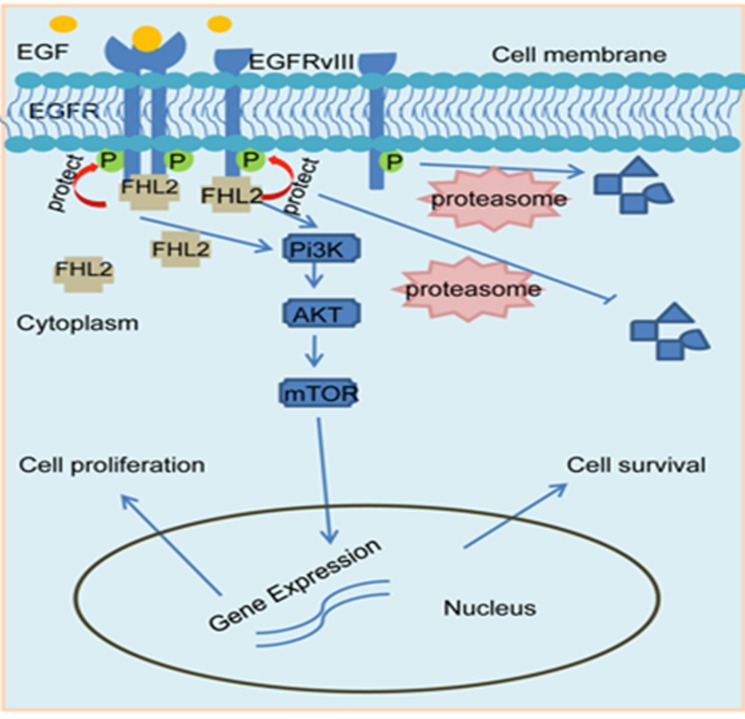
FHL2 increases EGFR stability in GBM cells FHL2 physically interacts with wtEGFR and EGFRvIII to increase their protein stability, which in turn further activates the downstream Pi3K-AKT signaling cascade.

Given the fact that both EGFR and FHL2 play a key role in GBM development, our recent paper investigated if there is a relationship between these two proteins [8]. We showed that expression of FHL2 is able to increase EGFR and EGFRvIII protein levels and this was due to an increase in protein stability rather than an increase in EGFR mRNA expression. In deed, we found that FHL2 physically interact with both wtEGFR and EGFRvIII to protect them from protein degradation in GBM cells, and this association is independent of the kinase activities of EGFR. As a result, the interaction between FHL2 and EGFR or EGFRvIII further triggers the activation of the downstream signaling pathways such as Pi3k-AKT signaling cascade (Figure 1). On the other hand, disruption of the protein interaction by targeting FHL2 with RNA interference decreases the protein levels of EGFR and EGFRvIII and reduces AKT phosphorylation. Functionally, targeting FHL2 expression prevents EGFRvIII+ GBM cell growth by inducing cell apoptosis in vitro and in vivo, suggesting that FHL2 is essential for EGFRvIII-mediated GBM tumor growth. Furthermore, FHL2 silencing significantly delays the tumor progression and prolongs the survival time of mice with EGFRvIII+ GBM cells. These findings have clinical significance because FHL2 expression is positively associated with EGFR expression at the protein levels in GBM samples from patients [8]. Therefore, our findings demonstrate a novel mechanism by which FHL2 regulates EGFRvIII-promoted GBM tumor growth and development, and that targeting FHL2 might provide a new and alternative approach for EGFRvIII+ GBM treatment. We may utilize RNAi against FHL2 or design small molecule inhibitor/peptide to block the protein interaction of EGFRvIII and FHL2 together with EGFR inhibitor (gefitinib or erlotinib) to treat GBM with EGFRvIII expression.
